# Probing RNA structure and dynamics using nanopore and next generation sequencing

**DOI:** 10.1016/j.jbc.2024.107317

**Published:** 2024-04-25

**Authors:** Emma Bose, Shengwei Xiong, Alisha N. Jones

**Affiliations:** Department of Chemistry, New York University, New York, New York, USA

**Keywords:** RNA, RNA structure, RNA dynamics, chemical probing, oxford nanopore, next generation sequencing, read deconvolution, HIV, riboswitch, SARS-CoV-2, long noncoding RNA

## Abstract

It has become increasingly evident that the structures RNAs adopt are conformationally dynamic; the various structured states that RNAs sample govern their interactions with other nucleic acids, proteins, and ligands to regulate a myriad of biological processes. Although several biophysical approaches have been developed and used to study the dynamic landscape of structured RNAs, technical limitations have limited their application to all classes of RNA due to variable size and flexibility. Recent advances combining chemical probing experiments with next-generation- and direct sequencing have emerged as an alternative approach to exploring the conformational dynamics of RNA. In this review, we provide a methodological overview of the sequencing-based techniques used to study RNA conformational dynamics. We discuss how different techniques have enabled us to better understand the propensity of RNAs from a variety of different classes to sample multiple conformational states. Finally, we present examples of the ways these techniques have reshaped how we think about RNA structure.

RNA plays a critical role in the regulation of a myriad of biological processes, from gene expression to epigenetic regulation (1, 2, 3). The regulatory role of RNA occurs not just through the primary sequence but also through the secondary and tertiary structures they adopt (4, 5, 6, 7). These structures can be conformationally dynamic, sampling numerous structured states that are driven by thermodynamics, co- and post-transcriptional modifications, nucleic acid and protein binding, and even changes in temperature and ion concentration (4, 8, 9, 10, 11). Conformationally dynamic RNAs include many different classes of RNAs, such as riboswitches, microRNAs, precursor-messenger RNAs, and long noncoding RNAs (12, 13, 14, 15, 16, 17, 18, 19). These RNA molecules have been shown to undergo structural changes that play a regulatory role in biological processes and have also been associated with disease (10, 20, 21, 22).

The intrinsic dynamics of RNAs underlie RNA regulatory activity on an atomic level, which can be reflected through local, nucleotide-specific conformational changes, or more globally, through rearrangements of structured motifs. These spatially and temporally conformational states have been investigated using a variety of biophysical techniques, including nuclear magnetic resonance (NMR) spectroscopy (23, 24), single-molecule optical tweezer experiments (25, 26), fluorescence resonance energy transfer (FRET) experiments (27, 28), and cryogenic electron microscopy (cryo-EM) (29, 30). Where limitations arise in the aforementioned techniques (due to RNA flexibility and size, as well as their non-trivial application), chemical probing experiments have enabled us to study the structure and dynamics of nearly every class of RNA, as this approach is not limited to the size and flexibility of the molecule studied (31). In this comprehensive review, we delve into the latest advancements in chemical probing experiments, shedding light on their pivotal role in deciphering the conformational dynamics of diverse RNA classes. These encompass viral RNAs, noncoding RNAs, and riboswitches, each offering a unique insight into how RNA functions through the conformationally dynamic structures that it adopts. We also dissect the critical aspects of experimental conditions essential for the successful execution and analysis of these experiments, including chemical probe and reverse transcriptase selection, the choice of sequencing, and programs used for analysis. We conclude with a forward-looking perspective, exploring the future application of this approach in elucidating RNA conformational dynamics, and their broader implications in areas such as drug development.

## Chemical probing experiments and modification detection

**Figure 1:**
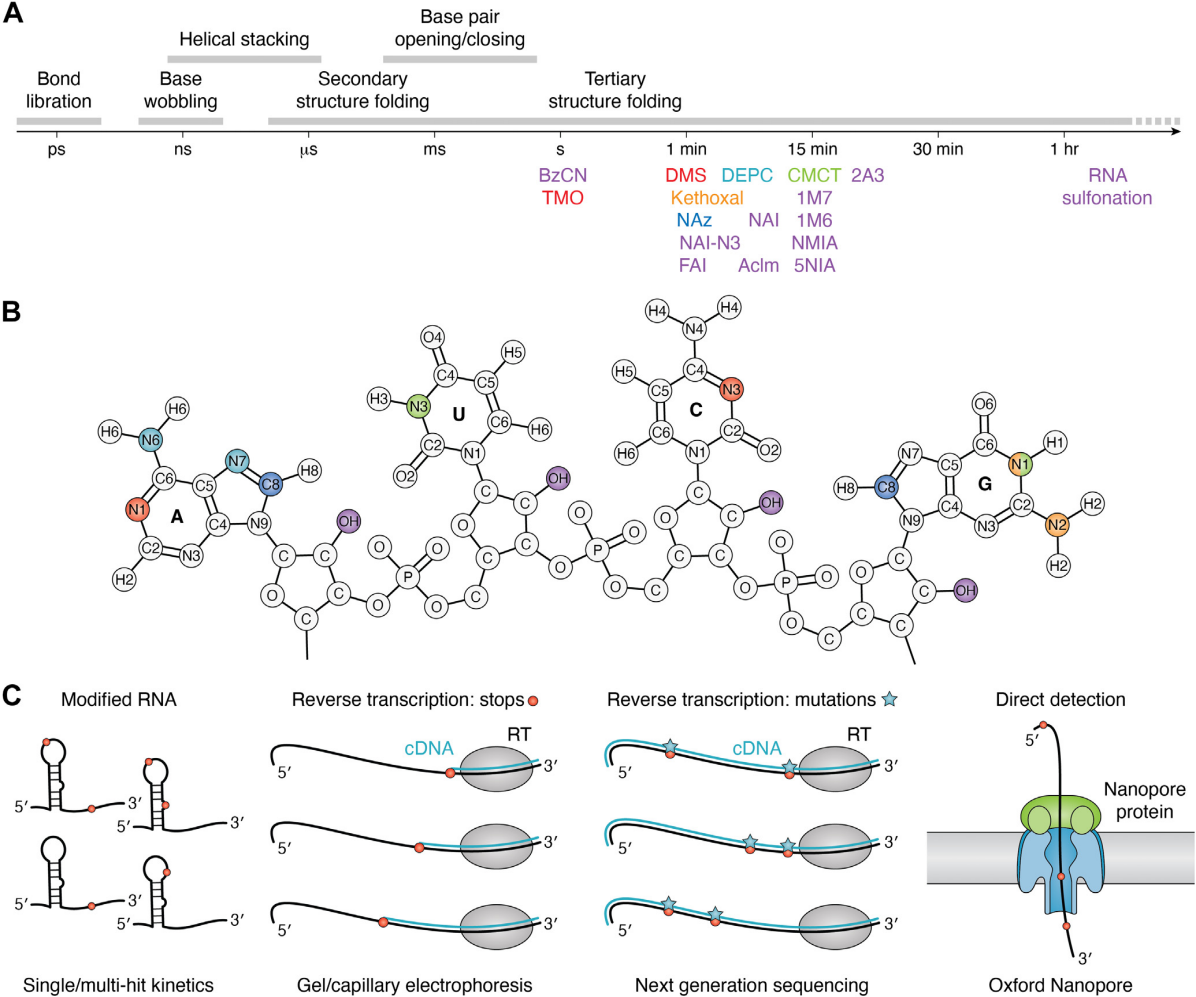
**RNA motions and chemical probe reactivity.***A*, time scale of various RNA structural motions and chemical probe reactivity. *B*, rate of modification for common chemical probes. Sites of chemical probe modification mapped to targeted atoms, color coded based on the colors of the text. See also Table 1. *C*, flexible nucleotides are targeted by chemical probes. Sites of modification can be detected through reverse transcription experiments, where modifications result in stalling of the reverse transcriptase or the introduction of a mutation. Modified nucleotides can also be detected directly through RNA Oxford Nanopore sequencing. 1M6, 1-methyl-6-nitroisatoic anhydride; 1M7, 1-methyl-7-nitroisatoic anhydride; 2A3, 2-aminopyridine-3-carboxylic acid imidazolide; 5NIA, 5-nitroisatoic anhydride; AcIm, 1-acetylimidazole; BzCN, benzoyl cyanide; CMCT, 1-cyclohexyl-(2-morpholinoethyl) carbodiimide metho-*p*-toluenesulfonate; DEPC, diethyl pyrocarbonate; DMS, dimethyl sulfate; FAI, 2-methyl-3-furoic imidazolide; NAI, 2-methylnicotinic imidazolide; NAz, nicotinoyl azide; NMIA, N-methylisatoic anhydride; TMO, trimethyloxonium.

In RNA chemical probing experiments, RNA (*in vitro* or in cells) is treated with a small chemical probe to identify flexible and single-stranded nucleotides. These probes react with the RNA on varying time scales, consistent with specific RNA motions (Fig. 1, *A* and *B*). While some chemical probes are base-modifying (such as dimethyl sulfate, DMS), others acylate the 2′hydroxyl group on the ribose ring, in an experiment known as “selective 2′hydroxyl analyzed by primer extension” (SHAPE) (Fig. 1*B* and Table 1). DMS is known to probe the flexible A and C nucleotides. However, it can also be conducted at a more alkaline pH (*in vivo*, cells are typically resuspended in a buffer with a pH of 8.3). This higher pH allows DMS to target all four bases, enabling the acquisition of more structural information (32, 33). To detect sites of RNA chemical probe modifications, reverse transcription (RT) reactions (stops and mutational profiling (MaP)) or third-generation sequencing (Oxford Nanopore Technology [ONT]) approaches are employed (Fig. 1*C*) (34, 35). Some of the more commonly used approaches include SHAPE-MaP and DMS-MaP-Seq; these, among others, have been thoroughly compared and reviewed (36, 37).

**Table 1:** Commonly used RNA chemical probes

Chemical probe	Experimental reaction time (37 °C)	Half-life (37 °C)	Target	Reference
Dimethyl sulfate (DMS)	Quenching based, 1–10 min	690 s	Flexible A (N1), C (N3), G (N7), and U (N3)	(111, 112, 113, 114)
1-cyclohexyl-(2-morpholinoethyl) carbodiimide metho-p-toluenesulfonate (CMCT)	Quenching based, 15 min	Quenching based, hour range	Flexible G (N1) and U (N3)	(113, 115, 116, 117, 118)
3-ethoxy-1,1-dihydroxy-2-butanone (kethoxal)	2.5 min	60 min	Flexible G (N1 and N2)	(113, 119, 120, 121)
Benzoyl cyanide (BzCN)	1 s	0.25 s	Flexible ribose (2′ OH)	(110)
2-methyl-3-furoic acid imidazolide (FAI)	5 min	73 min	Flexible ribose (2′ OH)	(122, 123)
2-methylnicotinic acid imidazolide (NAI)	10 min	Quenching based, 30 min	Flexible ribose (2′ OH)	(37, 122)
2-aminopyridine-3-carboxylic acid imidazolide (2A3)	20 min	Quenching based, 60 min	Flexible ribose (2′ OH)	(37, 101)
1-methyl-6-nitroisatoic anhydride (1M6)	15 min	31 s	Flexible ribose (2′ OH)	(37, 40)
2-(Azidomethyl)nicotinic acid imidazolide (NAI-N3)	5 min	30 min	Flexible ribose (2′ OH)	(124, 125)
1-methyl-7-nitroisatoic anhydride (1M7)	15 min	17 s	Flexible ribose (2′ OH)	(37, 126)
*N*-methylisatoic anhydride (NMIA)	15 min	260 s	Flexible ribose (2′ OH)	(37, 113, 122, 127)
5-nitroisatoic anhydride (5NIA)	15 min	∼100 s	Flexible ribose (2′ OH)	(37, 128)
RNA sulfonylation	24 h	90 min	Flexible ribose (2′ OH)	(128)
Nicotinoyl Azide (NAz)	3 min	ps time scale	Flexible G and A (C8)	(129)
TMO (trimethyloxonium)	30 s	7.5 s	Flexible A (N1) and C (N3)	(114)
DEPC (diethyl pyrocarbonate)	8 min	7 min	Unpaired A at N6 or N7	(53, 130)
AcIm (1-acetylimidazole)	9 min	3 min	Flexible ribose (2′ OH)	(90, 131)

Reaction times for SHAPE probes (Target: 2′OH) corresponds to ∼5× the half life.

During RT-stop experiments, reverse transcriptases stall at sites of modification during elongation, resulting in an accumulation of complementary DNA (cDNA) fragments that can be quantified by polyacrylamide gel electrophoresis or capillary electrophoresis (38, 39). During RT-MaP experiments, the presence of manganese chloride causes retroviral-derived reverse transcriptases (of note, group II intron-based reverse transcriptases do not require a manganese cofactor) to be error-prone; their fidelity is decreased and they will introduce a mutation in the cDNA (40, 41, 42, 43). Following amplification of the cDNA, these mutations can be detected through next-generation sequencing (NGS) platforms (*i.e.*, Illumina), and third-generation sequencing approaches (*i.e.*, Pacific Biosciences (PacBio), and ONT) (44, 45).

Oxford Nanopore Technology can also be used to directly evaluate modified RNAs. Nucleic acids are characterized in real time by recording the fluctuation of ionic current as the nucleic acid is passed through a nanopore protein (46). Relative to an unmodified RNA control, the presence of a covalently linked chemical probe impacts the dwell time and causes differential fluctuations in ionic current as nucleotides are threaded through the pore (47). Thus, nucleotides possessing chemical probe modifications can be identified (base-calling).

Reactivity profiles, whether based on the accumulation of cDNA fragments, the mutational profile arising from DNA sequencing approaches, or the direct detection of modifications in the RNA by ONT, are then generated to aid *in silico* secondary structure prediction (48, 49, 50).

## Identification of RNA structural ensembles from sequencing-based chemical probing data

With the development of NGS and ONT sequencing approaches to detect chemical modifications, it was realized that sequenced reads can be deconvolved and grouped according to their modification patterns or mutational profiles. Specifically, each read corresponds to a distinct structured state (and sometimes, a transitional state) sampled by the RNA. These reads can then be clustered based on the similarity of their profiles, thereby aiding secondary structure prediction of all structured states sampled by the RNA in solution. Notably, the number of identified clusters is correlated with the number of structures that comprise the structural ensemble. The proportion of reads in each cluster is also related to the population of a particular conformation in the ensemble.

Several algorithms have been developed to detect the various structured states of an RNA in solution based on sequenced reads from NGS and ONT experiments. In this review, we focus on RING-MaP (RNA interaction groups by mutational profiling) (51), DREEM (Detection of RNA folding ensembles using expectation-maximization) (52), DRACO (Deconvolution of RNA Alternative COnformations) (52, 53), DaVinci (Determination of the Variation of the RNA structure conformation through stochastic context-free grammar) (35), DANCE-MaP (Deconvolution and Annotation of riboNucleic Conformational Ensembles measured by Mutational Profiling) (32), and Single-Molecule Structural SEQuencing (SMS-seq) (54). RING-MaP, DREEM, DRACO, and DANCE-MaP are thermodynamically independent algorithms whereby sequenced reads are directly clustered without the need for thermodynamics-driven structure modeling (55). DaVinci and SMS-seq are thermodynamic-dependent algorithms that take into account the possible structures sampled by an RNA from a Boltzmann ensemble, and then sequenced reads are used to reweight the structured ensembles. Two excellent reviews of the various types of ensemble deconvolution based on NGS approaches were recently published (55, 56). Here, we provide a brief overview of NGS-based and ONT-based deconvolution programs and their applications and limitations.

### RING-MaP

RING-MaP (2014) is a thermodynamically-independent approach developed to deconvolute sequencing reads based on mutational profiles (51). RING-MAP involves the use of spectral decomposition techniques to analyze RNA secondary structures. The process begins with the construction of a graph where vertices represent RNA bases and edges signify co-mutational relationships based on experimental reactivity data. Following eigen-decomposition, the number of structural conformations making up the ensemble is determined from the number of Eigen-gaps (which is the distance between consecutive Eigen-values) exceeding an empirically defined threshold (0.03 by default). Reads are then assigned to each conformation/cluster by K-means clustering. RING-MaP was verified with the *Escherichia coli* thiamine pyrophosphate (TPP) riboswitch, the *Tetrahymena* group I intron, and *Bacillus stearothermophilus* RNase P catalytic domain RNAs. One of the drawbacks of RING-MaP is that being K-means a hard clustering method, reads can only belong to one cluster. This can lead to incorrect reconstruction of the reactivity profiles and under-estimation of cluster abundances. Further deconvolution methods, such as DREEM, DRACO, and DANCE-MaP were designed to address these issues.

### DREEM

DREEM (2020) is a thermodynamic-independent approach that utilizes an expectation maximization (EM) algorithm to group reads (from DMS-MaPseq (Mutational Profiling with Sequencing) experiments) with similar mutational profiles (34). It involves aligning raw sequencing data to a specific RNA sequence, converting aligned reads into binary “bit vectors” representing mutations, and then clustering these vectors based on mutational profiles using an EM algorithm. A default of two clusters is set for each analysis; a Bayesian Information Criteria (BIC) test is used to validate whether two or more clusters are likely to be present. The stringency of the BIC test, however, can lead to an under-estimation of clusters. Where reads do not fit with a majority of reads from another cluster, a penalty is incurred, causing a failed BIC test, and new clusters are not promoted. To save on computational cost, a maximum of four clusters can be detected by DREEM, though the cap is set to 2 by default, which may also lead to an overestimation due to the bayesian information criterion. DREEM was validated by mixing two RNA molecules (ribosnitches) that exhibit high sequence similarity, but form different structures, based on the RNA transcribed from the *MRPS21* gene (human), as well as with the *Vibrio vulnificus* adenine deaminase (*add*) riboswitch and with the RRE of HIV-1_NL4-3_; the latter two RNAs are discussed in more detail in the following sections.

### DRACO

RING-MaP, DREEM, and DANCE-MaP (see below) are only capable of identifying local conformational changes; this limitation arises due to current constraints of short Illumina read lengths. DRACO (2021) incorporates a sliding window approach to link the structural information across consecutive overlapping windows, hence enabling the use of information from tiling reads. The DRACO algorithm utilizes a customized fuzzy clustering approach with graph cuts to assign vertices to clusters based on their affinity. Being a soft clustering approach, reads sharing the same modification pattern can be assigned to different conformations/clusters. It then proceeds by merging compatible windows and re-assigning reads to refine the cluster analysis. While drawing inspiration from spectral clustering, the algorithm incorporates tailored steps and adaptations to effectively capture the structural diversity of RNA molecules from sequencing data. Additionally, DRACO is the only algorithm capable of automatically estimating the number of conformations, without the need to put a cap on the maximum number of clusters to prevent overestimation. However while it addresses the limitations of the aforementioned programs, it does not provide seamless data regarding the conformational ensemble of long transcripts (34, 53). This may lead to incorrect pairing of conformational states at the 5′ and 3′ ends. DRACO was validated both on a large dataset of *in silico*-simulated data, and on previously published chemical probing datasets for *E. coli cspA 5′* UTR, *V. vulnificus* adenine deaminase riboswitch, SARS-CoV-2 RNA, and the HIV-1 RRE.

### DANCE-MaP

DANCE-MaP (2022) also uses an EM algorithm to group reads from DMS-based experiments (32). A machine learning (ML) framework that fits single-molecule reads to multiple reactivity profiles is used to increase the number of fitted states successively until the optimal solution is found. DANCE-MaP categorizes RNA bases into active and inactive based on whether their background-subtracted mutation frequency exceeds 0.2% or not, thus preventing highly mutable positions from skewing the analysis. The method conducts separate deconvolution processes for active and inactive positions, allowing for a nuanced exploration of structural characteristics. Initially, reactivity data for active positions is deconvolved to determine cluster proportions, followed by a second deconvolution of the reactivities for inactive positions. These are clustered separately to prevent specific positions from dominating the clustering phase. This is accomplished through the incorporation of RING and PAIR correlation analyses that enhance the ML framework to detect tertiary structural interactions and specific base pairing. Finally, DANCE-MaP can deconvolute ensembles of up to five states with a minimum population of 5%. DANCE-MaP was benchmarked with the *V. vulnificus add* adenine riboswitch both in the absence and presence of ligand, as well as with the 7SK ncRNA.

### DaVinci

DaVinci (2022) was designed to account for limitations arising from short Illumina read lengths. PacBio, which can sequence up to 25 kilobases, is used to sequence DNA reads following chemical probing of the RNA (35). With DaVinci, a stochastic context-free grammar (SCFG) algorithm is used to determine the structural conformation of each RNA molecule by directly measuring the percentage of clusters by counting each single RNA structure derived from the probing data. The process involves converting sequencing reads into binary 'bit vectors' to represent mutations. Subsequently, an SCFG algorithm is used to derive RNA structures reflecting each mutation profile independently of thermodynamic parameters. These structures, encompassing mutation details and single-stranded nucleotides, are presented as production rules with logical “or” operations. Following transformation into a numeric matrix, dimensionality reduction techniques aid in determining representative RNA structures for each conformational cluster, facilitating analysis of RNA structure diversity and functional implications. DaVinci was benchmarked with class II *COOLAIR* isoforms, HIV-1 RRE61 RNA, and TenA RNA; additional published chemical probing data of the HIV-1 RRE and cspA 5′ UTR was also used for the validation.

### SMS-seq

As an alternative to PacBio, ONT experiments can also be used to assess the conformational dynamics of long transcripts. SMS-seq (2022) assesses the heterogeneity of RNA structural ensembles in two ways (54). By the first approach, all reads are individually folded *in silico* with modifications used as hard constraints. The number of reads resulting in the same structure is counted, representing the population of each structure in the solution. In the second approach, the RNAs are folded based on minimum free energy (MFE), maximum expected accuracy (MEA), and ensemble centroids. SMS-seq reads are then grouped using a binary distance metric. The group is defined by the proportion of reads in which both positions are modified relative to the number of reads in which at least one is modified. SMS-seq was benchmarked using the riboswitches *F. nucleatum* FMN and thiamine pyrophasphate and with synthetic RNAs. Additionally, ncRNAs (snR37 and snR17a), non-poly(A) RNA, endogenous RNA, rRNAs, and isolated poly(A) RNA from *Saccharomyces cerevisiae* (yeast) were used to investigate the ability of SMS-seq to unveil the structural landscape of the transcriptome.

Whether by DNA or RNA sequencing approaches and notwithstanding their respective limitations, deconvolution algorithms have provided valuable insight into the conformational dynamics of RNA and the regulatory role of dynamics in governing biological processes. Following the deconvolution of RNA structures with these approaches, it is important that other experimental techniques are carried out to further validate the structural ensemble. These techniques include, for example, native gel analysis (whereby different conformational states migrate differently through a gel), and the introduction of modest mutations to lock specific structured states. Nevertheless, the structural ensembles of over 30 RNAs have been investigated to date using deconvolution approaches. While a previous review has highlighted the results of a few of these RNAs, we expand upon this by comparing the structural ensembles that have been studied using overlapping approaches and highlighting a few additional dynamic RNA structures investigated through ONT (56).

## Structurally dynamic RNAs investigated by overlapping approaches

### Viral RNA elements

#### Human immunodeficiency virus

The human immunodeficiency virus (HIV-1) is a lentivirus that attacks and destroys CD4 cells, compromising an infected patient's immune response (https://www.who.int/data/gho/data/themes/hiv-aids). Since the start of the HIV epidemic, approximately 40 million people have died from HIV-1 (https://www.who.int/data/gho/data/themes/hiv-aids). The life cycle of the virus involves the transmission of viral RNA to a host cell, the reverse transcription of the RNA into DNA, followed by the integration of the viral DNA into the cellular genome. The DNA is then transcribed by the host cell to produce more viral RNA that encodes for viral proteins; together, viral RNA and proteins are packaged and exported to infect other cells.

#### HIV-1 RRE

**Figure 2:**
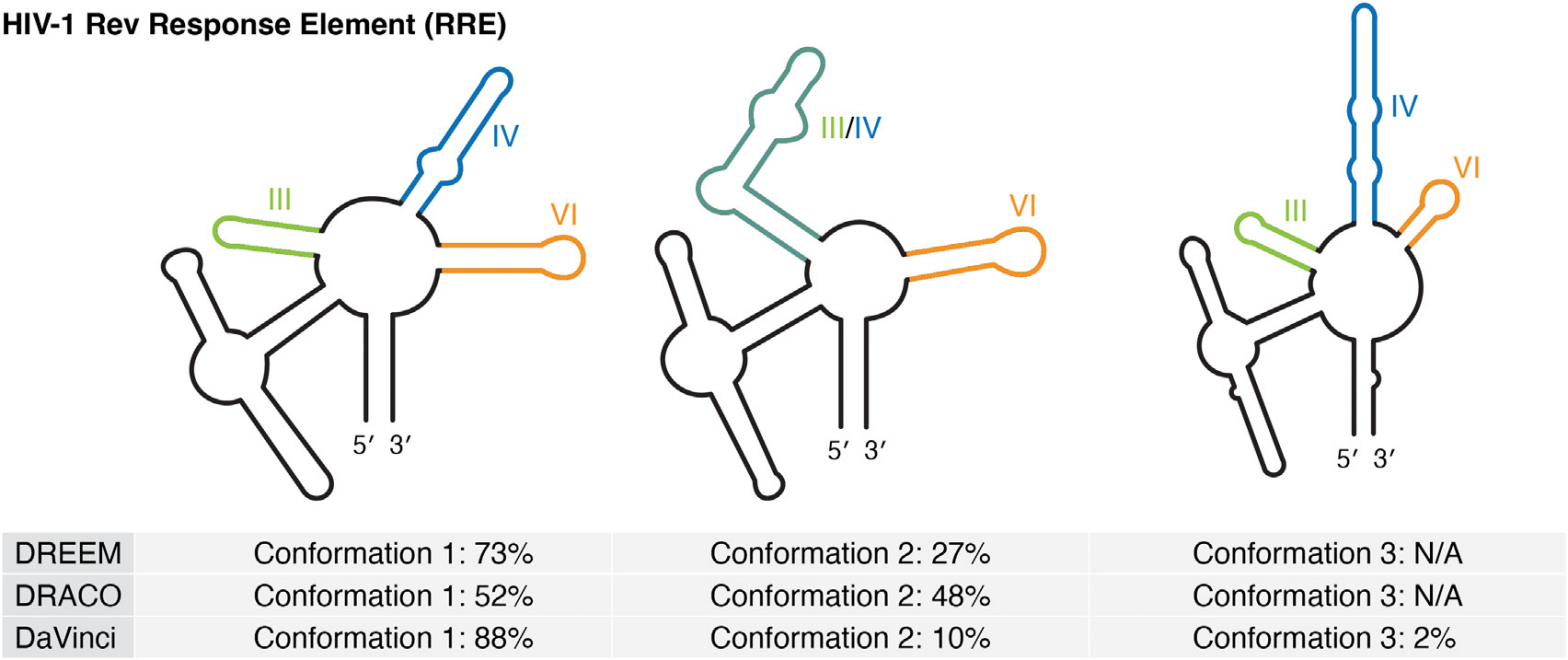
**The HIV-1 RRE genomic RNA is conformationally dynamic.** DREEM and DRACO deconvolution reveal two conformations. DaVinci deconvolution of HIV-1 RRE predicts three conformations. Population percentages for each approach are listed. Chemical probing was carried out with DMS; DMS data was re-analyzed in DRACO and DaVinci analyses.

The Rev protein recognition element (RRE) is a 350-nucleotide RNA element that is responsible for the export of HIV RNA. Here, the Rev protein binds the RRE RNA to facilitate its transfer from the nucleus to the cytoplasm (57). The HIV-1 RRE has been reported to sample two structures in solution at near equimolar concentrations (58, 59). Three deconvolution approaches, DREEM, DRACO, and DaVinci, have been used to investigate the conformational heterogeneity of the HIV-1 RRE (Fig. 2) (34, 35, 53). It is noteworthy that DRACO and DaVinci analysis were performed on the DREEM-based probing data. DREEM and DRACO deconvolution analysis identified two conformations of the HIV-1 RRE, while DaVinci deconvolution analysis revealed three. Although they vary in population abundance, two of the three DaVinci-predicted structures are in good agreement with the two structured states identified by DREEM and DRACO. In the major conformation, five stem loop structures are formed: SL I, II, III, IV, and IV. DREEM estimates this conformation makes up 73% of the structural ensemble, whereas DaVinci reports an 88% population abundance. DRACO analysis reveals a 52% abundance for the major conformation. Conformation 2, the minor conformation, makes up 27% of the DREEM structural ensemble, but was reported to be present at 10% by DaVinci. This conformation is represented at 48% by DRACO. Here, SL III and IV are combined to form a new SL (III/IV) structured domain. The third, hidden conformation, representing 2% of the structure ensemble, was identified with the DaVinci approach; stem loops III, IV, and V are reorganized to form a new, elongated structured domain.

The HIV-1 RRE's conformational flexibility is closely tied to its functional activity in viral replication (58, 60). Different conformations of the RRE can modulate HIV replication kinetics by altering the nature of the complex that is formed with the Rev protein, and effectively regulate the rate by which the virus replicates. Though both conformations are bound by Rev, conformation 1 has been linked directly to increased replication (61). The biological relevance of the third structured state may represent a transitional state that exists between conformation 1 and 2, but this has yet to be thoroughly investigated (61).

An interesting question, then, is why the third conformation was not detected by DREEM or DRACO. Considering that DaVinci analysis was performed on the same dataset analyzed by DREEM, the regimes by which these two algorithms function are the most likely differentiating factor. One possibility is that the default cluster limit was set to 2 in DREEM, preventing the realization of the third cluster. Alternatively, to avoid read cluster overfitting, DREEM uses an implementation of BIC testing. Reads that cannot be clustered can be described as limited or inadequate in representing the data; they may not effectively capture the underlying reactivity patterns in the data. Effectively, these clusters may be less reliable or informative in explaining the relationships among the reads. Where DREEM clusters read based on the similarity of chemical modification and are limited by stringent BIC tests, DaVinci directly clusters the RNA structures derived from the mutation profile of each single RNA molecule; reads are not discarded from the analysis. DaVinci uses the modifications in each read to hard-constrain a separate structure prediction, meaning that most structure models are constrained by at most 1 to 3 modifications, which might introduce significant biases.

Nevertheless, all approaches reveal that the HIV-1 RRE is conformationally dynamic, offering insight into its ability to function to regulate viral replication. Surprisingly, it was observed that the RRE sequence consistently adopted the same alternative structures, whether in an *in vitro*, *in vivo*, or in a virion environment, with a preference for conformation 1. These findings suggest that the variations in RRE's secondary structures are primarily influenced by the inherent thermodynamics of the RNA rather than specific features of the cellular environment.

#### Severe acute respiratory syndrome coronavirus 2

The severe acute respiratory syndrome coronavirus 2 (SARS-CoV-2) is an enveloped, positive sense, single-stranded RNA virus that infects the respiratory system of mammalian and avian species (62). The replication process of SARS-CoV-2 involves the attachment, fusion, and entry of coronavirus particles into a cell (63). Following entry, the viral genomic RNA is released from the viral particle; it is immediately translated into protein *via* two open reading frames: ORF1a and ORF1b (64). The resulting polyproteins are processed into individual proteins to form the viral replication and transcription complex. Newly produced genomic RNA is assembled into new particles that are released from the cell to infect other cells. Since the start of the SARS-CoV-2 epidemic in 2020, nearly 7 million deaths have been attributed to the virus (https://covid19.who.int/). Understanding the conformational space sampled by the viral RNA could lead to alternative therapeutic targets; both DREEM and DRACO have been used to investigate the conformational heterogeneity of SARS-CoV-2 viral RNA.

#### SARS-CoV-2 FSE

**Figure 3:**
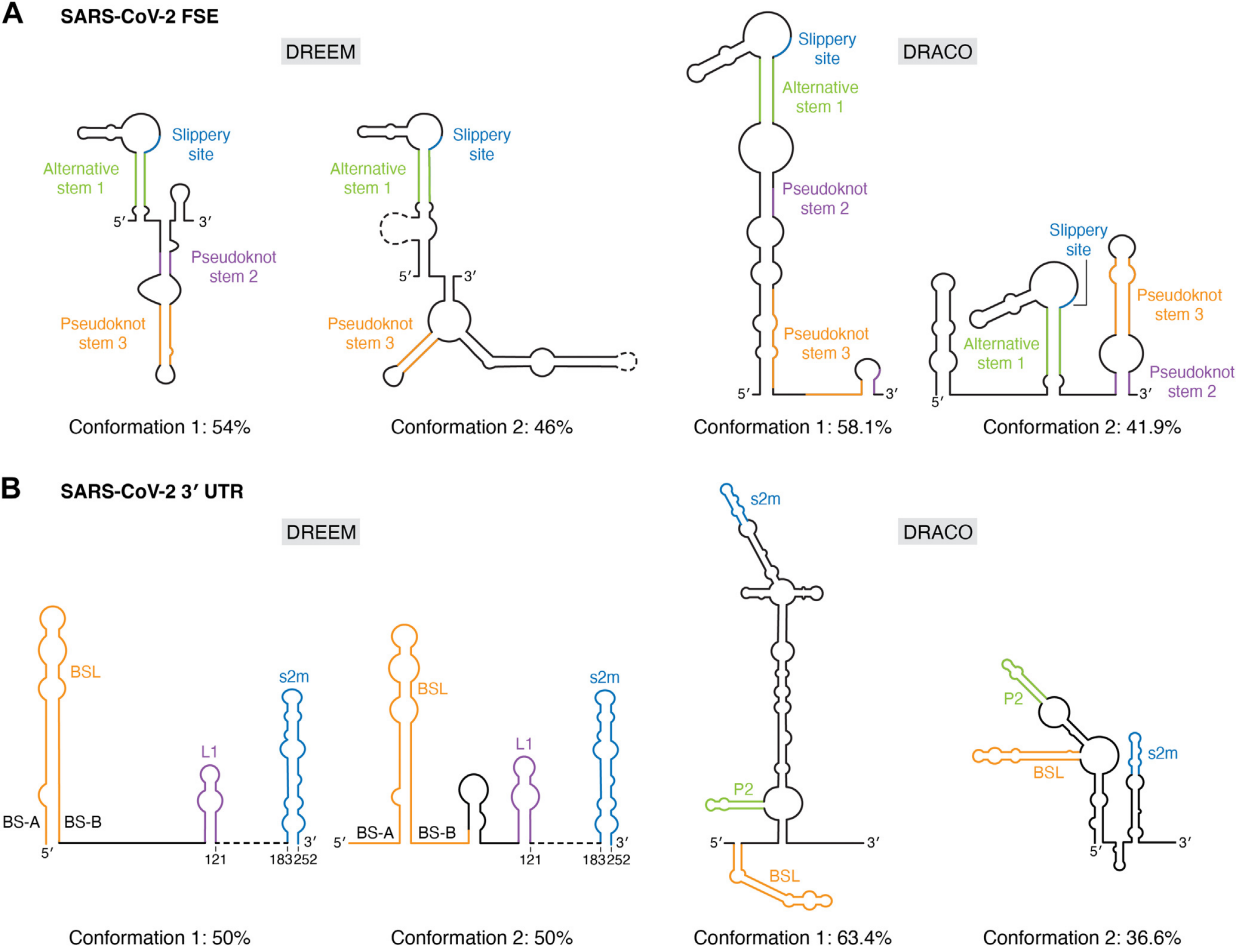
**The SARS-CoV-2 viral RNA is conformationally dynamic.***A*, the SARS-CoV-2 FSE adopts four different predicted structures as determined by DREEM (54% and 46%) and DRACO (58.1% and 41.9%). These deconvolved structures are from DMS probing experiments. *B*, the SARS-CoV-2 3′UTR samples four conformations as determined by both DREEM (50% and 50%) and DRACO (63.4% and 36.6%) deconvolution approaches using DMS.

The frameshift stimulating element (FSE) is a major drug target due to its role in ribosomal slippage about the ORF1a and ORF1b boundary (65, 66, 67, 68). Analysis of the conformational heterogeneity of the SARS-CoV-2 FSE by DREEM revealed that it samples two structured states (Fig. 3*A*) (65, 66, 67, 68). Conformation 1 is represented in solution at 54%, whereas conformation 2 is sampled 46% of the time. The major difference between these two structures is the absence of a locally folded helix, pseudostem 2, in conformation 2. A long-range stem is observed in place of pseudostem 2 in conformation 2; this long-range interaction promotes increased frameshifting in the FSE (conformation 1: 17%, conformation 2: 43%). The increased rate of frameshifting corresponds to increased production of viral protein from ORF1b.

The conformational heterogeneity of the FSE was also analyzed using DRACO, revealing two conformations (58.1% and 41.9%) (Fig. 3*A*) (53). These are moderately different from those predicted by DREEM. Conformation 2 of the FSE most closely resembles DREEM conformation 1. Here, the slippery site, alternative stem 1, and pseudoknot stems 2 and 3 are folded locally, with minor structural differences. The other conformation predicted by DRACO does not fold to form pseudoknot stem 2 or 3; instead, the nucleotides involving these helical domains are base paired with other regions in the RNA.

It is interesting that only one of the conformations partially overlapped between DREEM and DRACO. There may be two reasons for this: the first explanation is based on the conditions under which the RNAs were investigated. Different buffers were used to refold and chemically probe the FSE RNA: prior to chemical probing and DRACO analysis, the RNA was refolded in a 5× buffer containing 500 mM HEPES (pH 7.9) and 500 mM NaCl, whereas for DREEM the RNA was refolded in a 300 mM sodium cacodylate buffer with 6 mM MgCl_2_. It is well known that buffer composition can influence how an RNA folds (69, 70). It has also been proposed that the FSE is even more conformationally dynamic than anticipated by DRACO and DREEM analysis (71); perhaps each of the four structured states exists in solution; however, this hypothesis has yet to be experimentally validated. The second reason may be linked to the software itself: in an early version of the preprint reported by the Rouskin lab, the DREEM deconvolved structures of the SARS-CoV-2 FSE were very similar to those determined through DRACO, but in the published manuscript the newly reported structures include a kb-spanning long-range interaction that may influence the overall fold. Furthermore, manual integration of structural information is implemented in the current version of DREEM, which may also influence the outcome (67).

#### SARS-CoV-2 3′UTR

Transcription and replication of SARS-CoV-2 requires *cis*-acting regulatory elements located at the 3′ untranslated region (UTR) for the binding of the replication transcription complex (RTC) (72). It has been proposed that in the β-CoV genus, an equilibrium between a bulged stem-loop (BSL) and a pseudoknot acts as a molecular switch for the transcription of the 3′ subgenomic RNAs, and that this pseudoknot is essential for the binding of β-CoV RNA-dependent RNA polymerase (73). Using DMS-MaPseq, the structure of the SARS-CoV-2 3′UTR was investigated in both infectious virions and minigene-transfected cells (Fig. 3*B*) (74).

Secondary structure prediction suggested that the 3′UTR is identical in both the *in vivo* minigene-transfected cells and infectious virions. The DMS reactivities for the lower region of BLS (BS-A and BS-B) and L1 between in-virion and *in vivo* minigene structures, however, did not agree with the predicted structure. The in-virion structure showed low DMS activities on the BS-A segment, while the *in vivo* minigene structure showed medium-to-high DMS activities on BS-A and L1, thus leading to the question of whether the 3′UTR might in fact be in equilibrium between an extended BSL (E-BSL) and a pseudoknotted conformation in minigene-transfected cells. To answer the question, DREEM was employed. DREEM did not identify a cluster with DMS reactivities that supported a pseudoknot conformation; instead, it grouped the sequencing reads into two other structural clusters in a 1:1 ratio. This ratio was observed both in virions and in infected cells. The first structural cluster possesses two stem loops, BSL and L1. The second structure is composed of three stem loops, with the lower helix of BSL (BS-B) extended such that a new hairpin between BSL and L1 is formed. The absence of a pseudoknot formation in either cluster suggests that viral proteins, such as RTC, may influence the formation of the pseudoknot through an “induced-fit” model.

The 3′UTR was also investigated using DRACO both *in vivo* and *in vitro* (Fig. 3*B*). The predicted structures for both conditions are identical. DRACO revealed two conformations, both of which are supported by extensive covariation and by independent studies that used COMRADES and SPLASH to investigate the SARS-CoV-2 RNA structure and RNA-RNA interactions (71, 75). The major conformation (63.4%) has a hypervariable region (HVR), a stem-loop II-like motif (s2m), a bulged stem-loop (BSL), and a distal stem-loop (designated P2), while the minor conformation (36.6%) has an alternative two-way junction containing BSL and P2. The HVR is rearranged such that s2m is folded independently. Notably, BSL and P2 have been predicted to sample two additional but alternate conformations, A and B. This is attributed to the dynamics of the HVR.

The deconvoluted structures of the 3′UTR of SARS-CoV-2 RNA predicted by DRACO and DREEM share similar structures for BSL and s2m, however the overall conformation of the RNA analyzed by the two approaches are rather different, despite both being probed by DMS. This may in part be due to how the RNAs were probed; DREEM analysis was carried out on the RNA in cells, whereas DRACO analysis was carried out on RNA that was extracted from cells, refolded, and probed *in vitro*. The cellular environment may pose alternative factors (*e.g.*, protein binding, salt, pH, compartmentalization, or even abundance) that influence how the RNA is folded. Similar to the SARS-CoV-2 FSE RNA, the 3′UTR may also be highly dynamic, and each of the structures illustrated in Figure 3 may represent some fraction of the structural ensemble sampled by this RNA.

Altogether, deconvolution-based analyses of the 3′UTR and the FSE of SARS-CoV-2 reveal that this viral RNA possesses a high degree of conformational heterogeneity. Further investigation of the dynamics of this system, possibly in the context of the full-length RNA, are necessary to better understand how the structural dynamics associated with this RNA are linked to its biological function. The identification of each structured state could enhance our ability to target these regions therapeutically.

### Riboswitches

Riboswitches (or RNA switches) are *cis*-regulatory elements that regulate gene expression through conformational switches induced by a small molecule ligand binding partner (76, 77, 78). Several riboswitches have been investigated using deconvolution approaches (32, 35, 51, 54, 79). While some have served as benchmarks to validate the functionality of deconvolution approaches, others have been identified and validated through deconvolution algorithms. Here, we describe the adenosine deaminase riboswitch encoded by the *add* gene from *V. vulnificus* and the thiamine pyrophosphate (TPP) riboswitch from *E. coli*.

#### Adenine riboswitch

**Figure 4:**
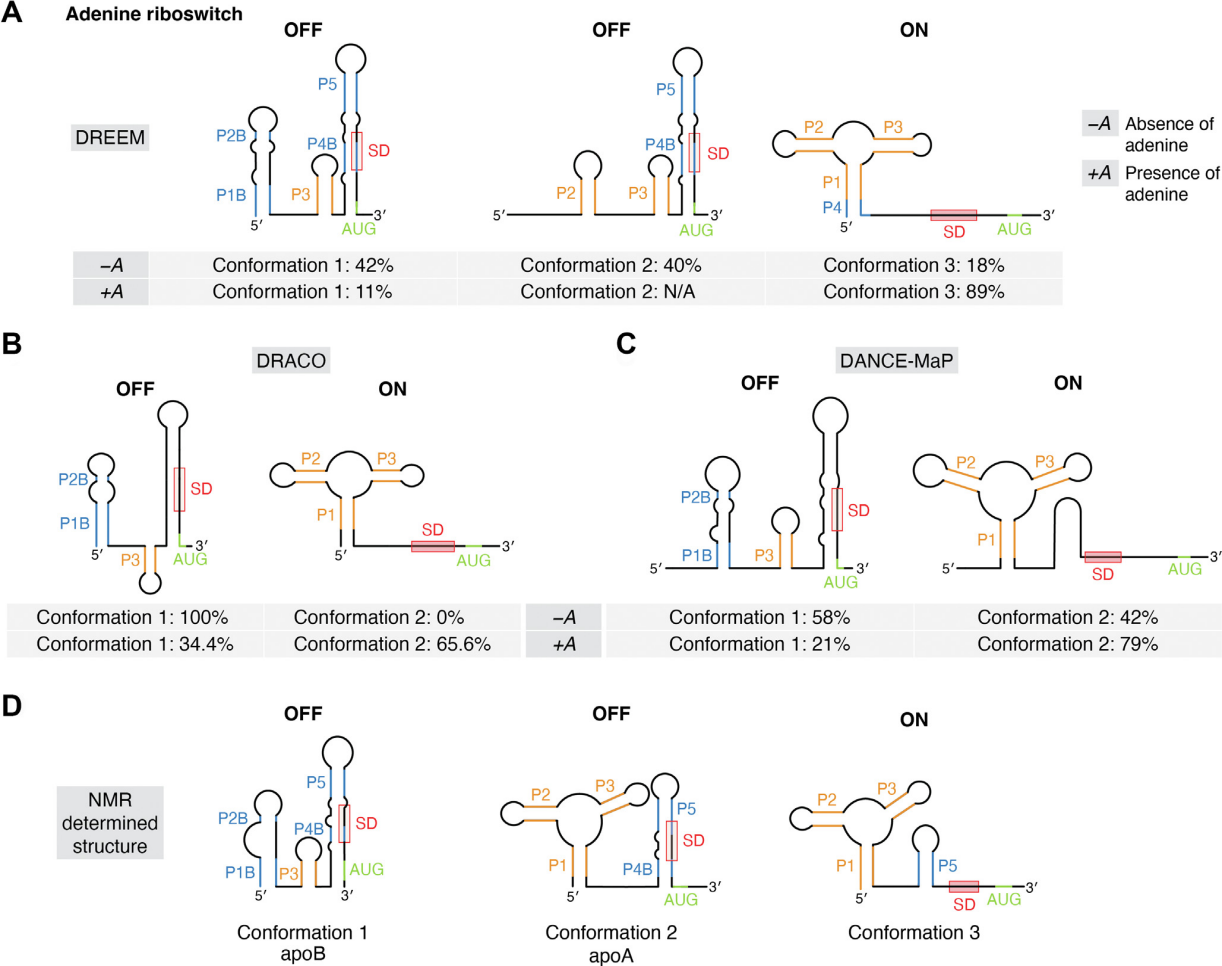
**The*****Vibrio vulnificus*****adenriboswitchisconformationallydynamic.***A*, DREEM-, (*B*) DRACO-, (*C*) DANCE-MaP-, and (*D*) NMR-determined structural ensembles in the absence and presence of adenine. Alternative structures of the three-way helical domains are shown in *yellow*, while other secondary structural motifs are shown in *blue*. The Shine Dalgarno (SD) and start codon (AUG) are also highlighted in *red* and *green* respectively. In all “OFF” structures, both SD and AUG are sequestered in a stem, while in the “ON” structures, they are single-stranded. DRACO and DANCE-MaP only detected one “OFF” structure, while DREEM detected two, agreeing with the NMR-determined structures.

The *V. vulnificus* adenine riboswitch is an ∼120 nucleotide (nt) RNA that controls gene expression; in the absence of adenine (‘OFF’ state), the Shine Dalgarno (SD) sequence, which serves as a ribosome binding site (RBS), is sequestered in the helical domain of a stem-loop. This prevents the ribosome from binding and translation therefore does not occur. In the presence of the adenine ligand (‘ON’ state), the structure of the adenosine deaminase riboswitch is modulated by the ligand to expose the RBS, promoting translation. Specifically, three helices termed P1, P2, and P3, form a three-way junction, and adenine binding near P1 destabilizes the P4 helix, resulting in the release of the AUG start codon and SD sequence (80). The adenine riboswitch has been evaluated by DREEM, DRACO, and DANCE-MaP (Fig. 4).

In the absence of adenine, DREEM uncovered three structured states, represented at 42%, 40%, and 18% (Fig. 4*A*). The higher populated conformations (42% and 40%) adopt structures that are structurally similar and both prevent translation due to a sequestered start codon, whereas the lesser populated population (18%) adopts a structure that allows for translation to occur due to the start codon being single-stranded. Interestingly, when adenine is added (5 mM), the percentage of the RNA sampling “translation ON” structure increases to 89%.

In the absence of adenine, DRACO identified a single conformation, which is not in agreement with DREEM and DANCE-MaP (see below). Conversely, upon the introduction of adenine, a notable conformational transition was observed, wherein approximately 65.6% of the RNA molecules shifted towards an orientation conducive to translation initiation. Concurrently, the remaining 34.4% retained the translation-inhibitory conformation, as substantiated by their pronounced correlation with the adenine-free control (Fig. 4*B*).

DANCE-MaP deconvolution reveals that in the absence of adenine, 42% of the structures in solution represent the translation ‘ON’ state, whereas 58% represent the translation ‘OFF’ state (Fig. 4*C*). With increasing concentration of adenine, the ‘ON’ state increases, and the ‘OFF’ state decreases. Even at 500 μM adenine, 21% of the structures still correspond to the ‘OFF’ state.

The structures sampled by the *V. vulnificus* adenine riboswitch in the absence and presence of adenine have previously been investigated with NMR (81). In the absence of ligand, the adenosine riboswitch surprisingly samples two distinct conformations (termed apoA and apoB; conformation 2 and 1 respectively in Fig. 4). Both contain a restrained Shine-Dalgarno motif, thus inhibiting translation, however the start codon in apoA but not apoB is single-stranded. Additionally, only apoA is able to bind adenine as in the apoB conformation the three-way junction is sequestered. These conformations are in equilibrium, and upon the addition of adenine, a third conformation is sampled by apoA. The active conformation and apoA are structurally similar, but apoA has a modulated ligand-binding core and only partially formed P1 and P4 helices.

Compared to the deconvoluted structures, each algorithm successfully determined the “ON” state whereby a three-way junction and an exposed start codon and SD sequence match the NMR-determined structure. Neither deconvolution algorithm detected an intact hairpin near the AUG start codon and SD sequence, as seen in the NMR structure of the “ON” state. With regards to the “OFF” structured state, there are some discrepancies with the “OFF” structured states: only DREEM detected two “OFF” conformations, whereas DRACO and DANCE-MaP deconvolution revealed one. The absence of additional conformations with DRACO deconvolution has been attributed to over-clustering, despite data from other biophysical and biochemical approaches suggesting at least two conformations exist in the solution (79).

#### TPP riboswitch

The *E. coli* TPP riboswitch is a 79 nt RNA that controls gene expression; in the presence of TPP (‘OFF’ state), the Shine Dalgarno (SD) sequence, which serves as a ribosome binding site (RBS), is sequestered in the helical domain of a stem-loop (82). This prevents the ribosome from binding and translation therefore does not occur. In the absence of the TPP ligand (‘ON’ state), the structure of the TPP riboswitch is modulated by the ligand to expose the RBS, promoting translation.

The conformational states of the TPP riboswitch, and their functional relevance have been extensively studied. RING-MaP revealed that in the absence of a ligand, two conformations coexist in solution (51). The major conformation (83%) corresponds to a state where the SD signal is single-stranded and accessible to the ribosome. The minor conformation (17%) has a reactivity profile that corresponds to a TPP-bound state, where the SD sequence is sequestered in structure. In the presence of the TPP ligand, three conformations were observed: a major conformation (75%), representing the “OFF” state, and two minor conformations, representing an “ON” state (20%) and an indecipherable state sampled by 5% of the RNA molecules in solution. Here, DMS reactivity was too low to accurately generate a chemical probing profile (51).

The conformational ensemble of the TPP riboswitch (in the presence of TPP, 6-fold excess) was also investigated using SMS-Seq (RNA was treated with DEPC), where two conformations were identified (54). Though population percentages were not reported, the predicted structures are in agreement with the previously reported crystal structure and RING-MaP analysis (83).

### Noncoding RNAs

#### COOLAIR lncRNA

Plant flowering is controlled by a collection of cis-acting lncRNAs known as COOLAIR that modify histones directly and help to create the epigenetic memory needed to control plant development (84, 85). The structure of COOLAIR (from five species) was chemically probed by SHAPE and CMCT, though no conformational dynamics were reported for the RNAs (84). In a recent study, the conformational dynamics of COOLAIR were extensively studied, revealing that its structural ensemble is directly related to its flowering function (35).

**Figure 5:**
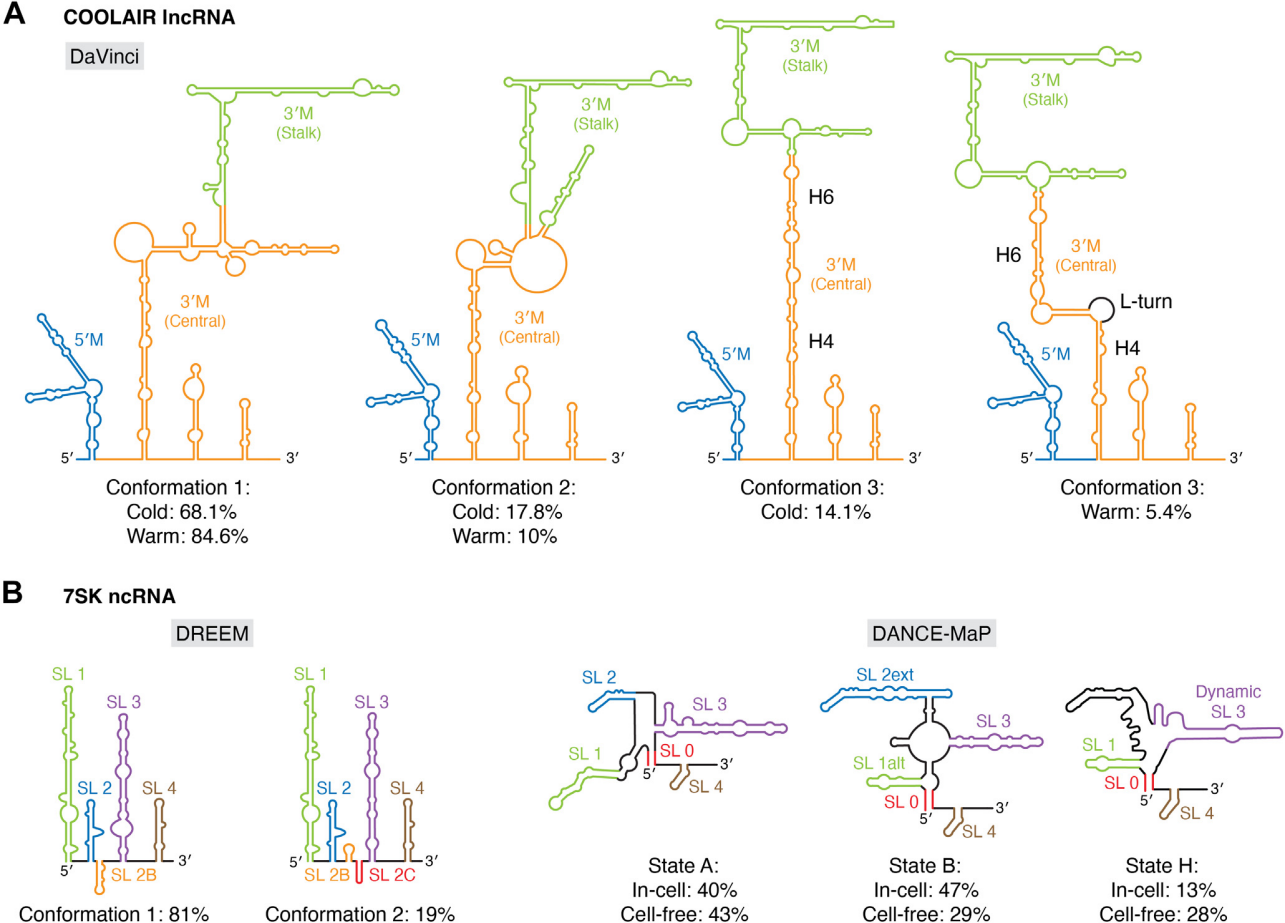
**Conformation dynamics of noncoding RNAs.***A*, the conformational dynamics of the COOLAIR lncRNA. COOLAIR adopts three conformations in cold and warm environments, as determined by DaVinci analysis. The significant structural difference is the L-turn between H4 and H6 in conformation 3, where in cold environments this turn is absent and instead, H4 and H6 are connected in a long-stem element. The population percentage of each conformation is listed in the figure. Probing was carried out with the NAI chemical probe. *B*, 7SK ncRNA possesses two conformations (81% and 19%) *via* DREEM. The structural differences are localized to SL2B, SL2C, and the base of SL3 in minor conformation. Two major conformations of 7SK are identified by DANCE-MaP. State H is an on-pathway intermediate. Population percentages are indicated in the figure.

The *in vivo* RNA structure of the COOLAIR antisense transcript was investigated using DaVinci analysis followed by NAI chemical probing experiments (Fig. 5*A*). Three major structural conformations of COOLAIR class II at the Arabidopsis floral-repressor locus FLOWERING LOCUS C (FLC) were identified under warm conditions. Warm conformation 1 makes up 84.6% of the total population of class II isoforms in warm environments. Warm conformation 2 (10%) and warm conformation 3 (5.4%) are the other two conformations. Three domains can be found in *in vivo* structural conformations, including the 5′ domain in exon 1; the 3′ major domain (3′M) or the central domain in exon 2; and the 3′ minor domain (3′m) or the stalk domain in exon 2 (35). In cold-grown plants, cold conformation 1 (68.1%), cold conformation 2 (17.8%), and cold conformation 3 (14.1%) are the three class II isoform conformations that have been identified. Although their relative proportions have shifted slightly, warm conformations 1 and 2 and cold conformations 1 and 2 are structurally identical. The section between H4 and H6 is connected and forms a long stem in cold conformation 3, differing from warm conformation 3, which contains an L-turn. In conclusion, class II isoforms have two main structural conformations, the proportions of which shift when the temperature decreases, and cold-grown plants develop a new conformation. These data indicate that RNA conformational changes play a vital role in the reaction of plants to their environment. Indeed, trapping the RNA into specific structured states influenced flowering in plants (35).

#### 7SK snRNA

7SK is an intricately structured 332 nt noncoding RNA that regulates the transcription activity of RNA polymerase II through interactions with a variety of proteins including HEXIM1/2, MePCE, LARP7, and hnRNP A1/A2 (86). A stem-loop structured element local to the 5′ end of the 7SK transcript, SL1, is bound by the HEXIM1/2 dimeric protein. HEXIM recruits the positive transcription elongation factor (P-TEFb) protein complex; this binding interaction inactivates P-TEFb (87). In its inactive state, P-TEFb is incapable of promoting elongation of nascent transcripts by RNA polymerase II. 7SK snRNA associates with hnRNP A1/A2, which prevents HEXIM1/2 from reassociating with P-TEFb. The ability for 7SK to bind to numerous proteins suggests that its structure changes upon protein binding. Indeed, previous reports using SHAPE-based experiments found that 7SK snRNA can adopt multiple structures (32).

DMS-MaPseq followed by DREEM analysis revealed that 7SK is present in at least two different conformations, with specific differences localized to the linker region connecting stem loops 2 and 3 and in the base of stem loop 3 (Fig. 5*B*). The major conformation (81%) has a minor stem loop (SL2B) in the linker region and an additional internal loop at the base of stem loop 3, whereas the minor conformation (19%) has two minor stem loops in the linker region and does not have the internal loop at the base of stem loop 3. Given that the characterization of 7SK snRNA alternative conformations was done in the absence of proteins, these results suggest that the distinct conformations act as scaffolds to assemble multiple RNP complexes, and that these binding events can further induce changes to 7SK snRNA architecture.

DMS chemical probing, followed by deconvolution analysis by DANCE-MaP, revealed that 7SK adopts three structured states, A, B, and H (Fig. 5*B*). Probing was carried out in cells and on RNA extracted from Jurkat cells (denoted cell-free). Despite minor changes in abundance levels, the structures are in excellent agreement. In-cell state A, represented at 40%, contains six structured regions: SL0, SL1, SL2, SL3, SL3a, and SL4. The same structure is observed in the cell-free state at a population of 43%. State B, containing the SL1alt stem, rather than SL1 (state A), is represented at 47%. SL2 is extended (SL2ext), and SL3a is no longer present. This structure makes up 29% of the structural ensemble under cell-free conditions. In state H, only SL1alt is stably folded; SL3 is highly dynamic. This structure comprises 13% and 28% of the structural ensemble in cells and in the cell-free state, respectively.

In state A, P-TEFb is bound to the RNA *via* SL1. In state B, where SL1alt is present, P-TEFb is released. The existence of SL1alt in state H indicates that state H, like state B, does not bind P-TEFb. A small antisense oligo (ASO-B) was designed to favor state B over state A by binding to nucleotides within the region between SL1 and SL2. The ASO caused the 7SK RNA structure to exclusively adopt state B, leading to release of P-TEFb and increased transcription of P-TEFb-responsive genes upon further transcriptional studies. This highlights the relevance of deconvolution algorithms in detecting biologically critical structured states in RNA.

The structures predicted for the 7SK snRNA by DREEM and DANCE-MaP are only modestly similar. There are several factors that may be at the root of these differences. First, the RNAs in each study were probed under different conditions. DREEM analysis was carried out on *in vitro*-transcribed RNA (100 mM sodium cacodylate, 140 mM KCl, 3 mM MgCl_2_, pH 7.5; TGIRT-III); DANCE-MaP was performed both *in vitro* (200 mM Bicine (pH 8.0), 200 mM KOAc (pH 8.0) and 5 mM MgCl_2_; SuperScript II), and in cells (human Jurkat cells; SuperScript II RNA extraction and *in vitro* folding (cell-free): human Jurkat cells; 37 °C; 200 mM Bicine (pH 8.0), 200 mM KOAc (pH 8.0), 5 mM MgCl_2_; SuperScript II). The absence of proteins may impact the structural landscape adopted by the RNA. Nevertheless, these analyses reveal the intrinsic dynamics of this transcript, which have been further supported by other approaches such as cryo-EM (88).

### Pre-mRNAs

#### SnR17a

**Figure 6:**
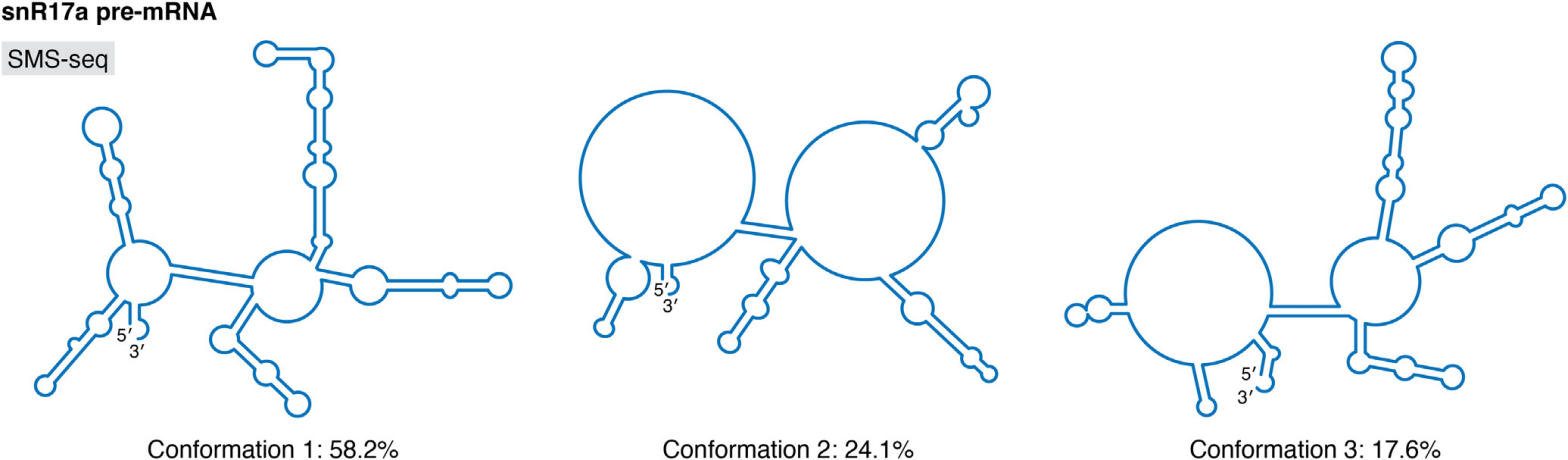
**snR17a conformational dynamics studied using SMS-seq.** Three structures were identified based on DEPC probing experiments; the percentage of each cluster is listed accordingly.

The small nucleolar RNA 17 (snR17a) is one of the most abundant RNAs in *S. cerevisiae* (89). snR17a is a homologue of the eukaryotic U3 snRNA, which is involved in early processing of the ribosomal RNA transcript (90). Given its binding activity with various protein factors, it has been proposed that U3-like RNAs do not adopt a single structured state (90). The snR17a RNA was chemically modified with DEPC and its conformational dynamics assessed using SMS-seq (Fig. 6). SMS-seq differs from other nanopore-based structure determination methods in that it is able to probe multiple bases on each unique RNA molecule and depict their conformational heterogeneity, which can reveal the different structures the RNA samples as well as the functionality of these differing structures. Three conformational clusters, where reads for each cluster represent 58.2%, 24.1%, and 17.6% of the total reads in solution, were identified with SMS-seq-based deconvolution. The major cluster (58.2%) adopts a structure similar to that previously described for the snR17 RNA (89). The second most abundant cluster (24.1%) is largely unstructured; 70% of the nucleotides are single stranded. The final cluster (17.6%) is structurally similar to the first cluster; however, nucleotides near the 5′ and 3′ terminal ends are unstructured. These latter structures have not yet been functionally explored, but investigation of their binding affinities with ribosomal processing proteins (*i.e.*, the U3 snRNP) may shed light on their respective functions.

### Perspective

Our ability to study the structure of all classes of RNA drastically improved with the coupling of chemical probing experiments with NGS and ONT sequencing (42, 45, 79, 91, 92). Structural probing experiments transitioned from studying single transcripts to genome-wide structural analyses (22, 92, 93, 94, 95, 96, 97). While it is true that conformational states of RNA could be studied using native gel electrophoresis or through the introduction of structure stabilizing or destabilizing nucleotide mutations (98, 99, 100), these are often time consuming and are not trivial to implement. The novel development of read deconvolution algorithms thus paved a way for the investigation of RNA conformational dynamics in a less cumbersome way, and have provided remarkable insight into the conformational dynamics of several different RNAs.

**Figure 7:**
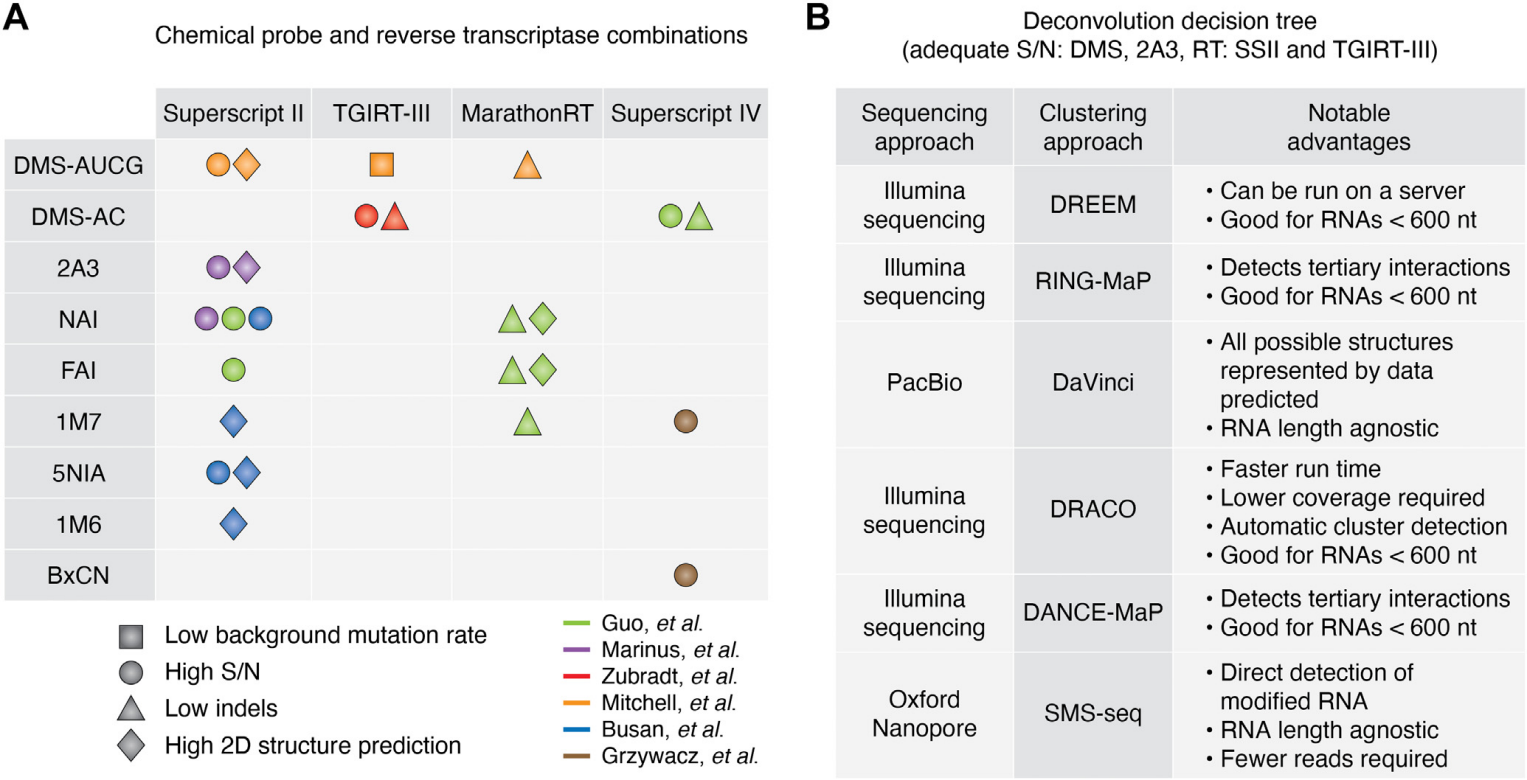
**Experimental design and preparation for deconvolution experiments.***A*, combinations of chemical probe and reverse transcriptase that have revealed low background mutation rates, high signal-to-noise, low indel counts, and high 2D structure prediction. *B*, DMS and 2A3 have demonstrated sufficient signal to noise for deconvolution. Each clustering approach has its advantages, based on the sequencing approach, RNA length, and other factors.

Determining which approach is most appropriate to study an RNA depends on several experimental factors, including the chemical probe, the reverse transcriptase, RNA length, and the anticipated dynamics of the RNA of interest. We discuss these herein and illustrate them in Figure 7.

#### Chemical probe and reverse transcriptase

Several comparative studies have been carried out to determine the most efficient probe for structure determination. Obtaining ideal and informative results is not limited to one probe, but depends on a combination of the chemical probe used and the reverse transcriptase employed (Fig. 7*A*). First, it is crucial for the probe to avoid having a bulky structure that could impede the processivity of the reverse transcriptase, a common issue observed with SHAPE probes. Second, not all probes can achieve adequate modification density; a good signal to noise ratio is important for the success of deconvolution algorithms. Though few studies have investigated the conformational dynamics of RNAs in living cells, it is noteworthy that mutation rates for chemical probes are typically lower when assessing the mutational profiles of RNAs probed in cells relative to cell-free experiments. Furthermore, across different cell lines, mutation rates induced by each probe vary (39, 101).

Four reverse transcriptases have been thus far demonstrated as feasible for mutational profiling experiments: SuperScript II (SSII), SSIV, and TGIRT-III RT (Thermostable Group II Intron Reverse Transcriptase), and MarathonRT. While each reverse transcriptase has led to the determination of numerous RNA structures, each has its own limitations. SSII, although the most conventional RT, has a background mutation rate nearly one order of magnitude higher than TGIRT-III (33, 102). Additionally, SSII, in the presence of manganese, incorporates multiple insertions or deletions (indels) when it encounters a modification rather than a single point mutation, making it non-trivial to assign the reactivity to the proper nucleotide. The other reverse transcriptases are also subjective to these mishaps, and can also pause at modification sites. MarathonRT has a lower background error rate relative to SSII (101). This impacts not only the signal-to-noise ratio but can affect the accuracy with which structures are predicted. MarathonRT and SSII must both be supplemented with manganese, although Marathon requires significantly less manganese (up to 2 mM for MarathonRT *versus* 6 mM for SSII) (101). TGIRT-III does not require a manganese cofactor. Each of these considerations is dependent on the probe used; a summary of the best probe/reverse transcriptase combinations is shown in Figure 7*A*.

#### RNA length and reverse transcriptase

Each reverse transcriptase has different processivity lengths. SSII has a low processivity rate beyond ∼800 nucleotides (103). Consequently, extracting structural information from extensively modified long RNA, such as messenger RNAs (mRNAs) and long noncoding RNAs (lncRNAs), is a highly arduous task, lest a randomer primer approach is used. TGIRT-III has a high processivity rate up through ∼1700 nucleotides (103). MarathonRT is the most processive up to ∼2500 nucleotides (101).

The length of the RNA should also be considered when selecting a sequencing and deconvolution approach (Fig. 7*B* and Table 2). Illumina-based approaches are excellent options for determining the structures of full length RNAs less than 600 nucleotides. For longer RNAs, Illumina-based approaches can also be used, but this is at the cost of combining shorter reads (150–600 base pairs) to obtain full-length RNA reactivity profiles. When considering deconvolution algorithms, it may be difficult to link conformational states samples at the 5′ end of the RNA with specific conformations sampled at the 3′ end. As aforementioned, DRACO employs a sliding window approach to circumvent this. Approaches that rely on PacBio (DaVinci) or ONT (SMS-seq) sequencing are more suitable for longer RNAs. The major advantages of PacBio and ONT over NGS are increased read lengths, minimized sequencing time, and reduced biases introduced to the sequence by PCR amplification (104, 105, 106, 107).

**Table 2:** Read lengths for Illumina, PacBio and ONT

Sequencing approach	Read length range
Illumina	150–600 bases
Pacific Biosciences	10–25 kilobases
Oxford Nanopore	10–2.273 megabases

#### Modification detection in RNA by ONT

ONT also has considerable limitations. First, because nanopore sequencing translocates RNA in a 3′ to 5′ direction, structural information near the 5′ end of samples is generally of lower quality. Second, both chemical modifications and naturally occurring RNA structure give rise to current signal and dwell time changes; differentiating between these two is a present challenge. Third, many of the commonly used chemical probes used in NGS and PacBio-based approaches (*e.g.*, DMS, NAI, 1M7, and NMIA) accentuate truncations in reads at sites of modifications, lead to mismatches, deletions, and insertions during base calling, and cause poor alignment (54, 79, 91). For example, cyclohexyl-3-(2-morpholinoethyl) carbodiimide metho-p-toluene sulfonate (CMCT) targets uracil bases and is miscalled as a cytosine. When compared to an unmodified RNA control that has been sequenced, this enables the detection of sites of modification. Similarly, DMS-modified cytosines are miscalled as uracils. NAI-N3-modified bases also lead to miscalling, and machine learning has been used to detect NAI-N3-modified bases (54, 79). Chemical probes that have been demonstrated as suitable for ONT-based approaches include NAI-N3, DEPC (diethyl pyrocarbonate), and AcIm (1-acetylimidazole) (54, 79, 91). DEPC, however, is limited in that it can only modify flexible adenosines. AcIm is capable of extracting information from all four bases and is small enough to avoid impacting motor processivity.

#### Coverage

In contrast to more conventional mutational profiling experiments, deconvolution algorithms require more reads and base coverage. Where approximately 5000 to 10,000 reads are necessary to determine the secondary structure of an RNA (42, 108), upwards of 50,000 to 100,000 reads are required to deconvolute the structural ensemble of an RNA using RING-MaP and DREEM (34, 51). Base coverage of at least 10,000× and 300,000× is necessary for adequate deconvolution by DRACO and DANCE-MaP, respectively (32, 53). ONT on the other hand, requires far fewer reads (several hundreds to tens of thousands) (54, 79, 91). This can be more costly, especially considering genome-wide analyses.

#### RNA length and conformational dynamics

Deconvolution algorithms are not designed to detect the dynamics of single nucleotides (*e.g.*, excited states), but rather can be used to assess larger local (or global) conformational changes. Significant changes in reactivity need to occur in order for clusters to be defined. As such, larger RNAs are excellent candidates for deconvolution algorithms because there is more opportunity to detect changes. Furthermore, deconvolution algorithms have not yet been designed to study the conformational equilibrium of rapidly fluctuating entities (*e.g.*, subsecond); in Illumina-based experiments, DMS is the most widely used probe, however, DMS reacts with RNA in a matter of minutes. While faster probes exist (*e.g.*, NAz and BzCN), these have not yet been utilized in deconvolution pipelines, but are suitable for mutational profiling experiments (Table 1). To ensure the success of these experiments, optimizing reaction conditions becomes crucial, promoting multiple probe binding events within the RNA molecule and enhancing the ability to accurately capture and analyze fast dynamics. If the rate by which the RNA transitions between structured states is unknown, time-resolved conformational analysis can be carried out to determine the most appropriate probe for capturing the structured states sampled by the RNA (109, 110).

With careful consideration, deconvolution algorithms serve as an excellent approach for investigating the conformational dynamics of RNA. Considering that chemical probing experiments are limited (*e.g.*, they do not reveal exact base pairs), and each deconvolution approach has its strengths and weaknesses, it is evident that further validating the results with other experiments is essential. These experiments include the introduction of mutations to validate specific conformations, adjusting the temperature during chemical probing experiments to shift the equilibrium of the structures, or even applying more high-resolution approaches such as NMR. Nevertheless, when pooled together and/or combined with other experiments, deconvolution approaches can shed light on the structural and molecular mechanisms that underlie ligand binding, and can also be used to identify and therapeutically target structured states of RNA that are implicated in disease.

## Conflict of interest

The authors declare that they have no conflicts of interest related to the contents of this article.
